# Developmental changes in the neural responses to own and unfamiliar mother's smiling face throughout puberty

**DOI:** 10.3389/fnins.2015.00200

**Published:** 2015-06-03

**Authors:** Tsunehiko Takamura, Shota Nishitani, Takashi Suegami, Hirokazu Doi, Masaki Kakeyama, Kazuyuki Shinohara

**Affiliations:** Unit of Basic Medical Sciences, Course of Medical and Dental Sciences, Department of Neurobiology and Behavior, Graduate School of Biomedical Sciences, Nagasaki UniversityNagasaki, Japan

**Keywords:** attachment relationship, development, near-infrared spectroscopy (NIRS), prefrontal cortex (PFC), puberty

## Abstract

An attachment relationship between boys and their mother is important for subsequent development of the ability to sustain peer relationships. Affective responses to attachment figure, especially mother, is supposed to change drastically during puberty. To elucidate the neural correlates underlying this behavioral change, we compared the neural response of boys at three different developmental stages throughout puberty to visual image of their own mothers. Subjects included 27 pre-puberty boys (9.0 ± 0.6 years), 31 middle puberty boys (13.5 ± 1.2 years), and 27 post-puberty boys (20.8 ± 1.9 years), and their mother's smile was video recorded. We measured their neural response in the anterior part of the prefrontal cortex (APFC) to their own mother's smile compared with an unfamiliar-mother's. We found that in response to their own mother's smiling, the right inferior and medial part of the APFC (Ch6) was activated in the pre-puberty group. By contrast, the left inferior and medial (Ch4) and superior (Ch2 and Ch5) APFC were activated in the middle-puberty group, which is presumably linked to empathic feelings fostered by memories of mutual experience with own mother. These findings suggest that different patterns of APFC activation are associated with qualitative changes in affective response to own mother around puberty.

## Introduction

The attachment relationship between mother and child is crucial for the ability to maintain an intimate relationship with others (Scaramella and Leve, 2004; Kriss et al., 2012). As an attachment figure, the presence of a care-giver, and in particular a mother, endows children with psychological security, and they seek physical and mental proximity to their mother for protection and emotional support when under threat (Bowlby, 1969).

During puberty, many maturational changes in physical appearance occur, which are derived from secondary sexual characteristics, changing relationships with family members, reorganization of sexual and social identity, and self-perception (Mendle et al., 2007). Throughout this period, the attachment relationship between a child and their mother goes through qualitative changes. Indeed, around puberty, children tend to spend more time with peers than family (Csikszentmihalyi et al., 1977; Steinberger, 1989) and are more concerned about maintaining peer relationships (Koepke and Denissen, 2012), and consequently become more independent from their parents (Fujisawa et al., 2012). Nevertheless, the mother continues to be an important figure. For example, deterioration of the maternal relationship during this period can increase the risk for emotional and behavioral problems in later life (Brumariu and Kerns, 2010).

Recently, several studies have identified neural correlates of positive affect that are induced in early childhood (de Haan and Nelson, 1997; Carver et al., 2003; Carlsson et al., 2008; Minagawa-Kawai et al., 2008; Nakato et al., 2011; Dai et al., 2014) by images of a child's own mother. Of particular relevance, Minagawa-Kawai et al. (2008) reported that infants show increased activation in the anterior prefrontal cortex (APFC), including the orbitofrontal cortex, in response to the smile of his/her mother, which presumably suggests that a mother's smile has rewarding value to her infant.

Importantly, recent studies have suggested that the mesolimbic reward circuit, which is strongly linked to the APFC, is reorganized throughout puberty (Todd et al., 2011). As already stated, developmental psychology literature has noted a qualitative change in the relationship between mother and child around puberty. On the basis of these findings, it is plausible to suggest that APFC activation to a mother's smile may change dynamically throughout puberty. However, most previous studies on maternal neural responses (de Haan and Nelson, 1997; Carver et al., 2003; Carlsson et al., 2008; Minagawa-Kawai et al., 2008; Nakato et al., 2011; Dai et al., 2014) have focused on early developmental stages (from early infancy to preschool years). Thus, despite the dramatic physiological and psychological changes that occur during puberty, to the best of our knowledge, no study has investigated the developmental course of maternal neural representation around puberty.

Hence, the overall aim of our study was to investigate the developmental course of APFC activation to a mother's smile. We examined different developmental stages throughout puberty, a time when the mother and child relationship changes markedly, as it is a period of rebelliousness during which attachment styles are altered. In addition, brain structures develop and are reorganized due to dynamic changes in sex steroid hormone levels during this period. Using near-infrared spectroscopy (NIRS), we compared PFC activation in response to a mother's smile in boys at three developmental stages. To exclude the confounding factor of facial familiarity, we adapted the experimental and analytic procedures reported in Minagawa-Kawai et al. (2008). Specifically, in each trial, neutral faces of the child's own mother and an unfamiliar woman were presented as baseline stimuli, and brain activation induced by the target smiling face was calculated by subtracting baseline oxyhemoglobin (oxyHb), which should reduce familiarity-related bias (Minagawa-Kawai et al., 2008).

We focused on the neural substrate of boys, because girls begin to show menstrual changes in sex steroid hormones after puberty. These developmental and menstrual hormonal changes in girls makes it difficult to control a number of parameters, and does not allow accurate assessment of developmental changes in APFC activation during puberty.

## Materials and methods

### Participants

A total of 102 healthy and naive pubertal boys participated (Table 1). The participants were classified into three groups: pre-, middle-, and post-puberty according to chronological age. The groups largely corresponded to Tanner stages (TS) (Marshall and Tanner, 1970; Fujieda, 1993), although sexual maturity was not assessed by the Tanner scale, which is based on physical examination. Pre-puberty boys were recruited from the 3rd grade of elementary school (approximately 9 years of age), and classified into the early stage of sexual maturity (approximately TS1). Boys at the 2nd and 3rd grade of junior high school (approximately 14 years of age) were recruited as middle-puberty boys, and classified into the middle stage of sexual maturity (approximately TS3). Boys at the 1st and 2nd grade of university (approximately 20 years of age) were recruited as post-puberty boys, and classified into the late stage of sexual maturity (approximately TS5). At all developmental stages, participants were recruited from several schools in different geographical areas of Nagasaki prefecture in Japan, representing students from a range of socioeconomic backgrounds. All subjects were right-handed on the basis of the Edinburgh Handedness Inventory (Oldfield, 1971). No participants suffered from any mental disorders or used medications that affect sex steroids or mental states. All participants and their parents gave written informed consent after being informed of the purpose of the experiment. The experimental protocol was in accord with the tenets of the Helsinki Declaration, and was approved by the Ethics Committee of the Nagasaki University Graduate School of Biomedical Sciences.

**Table 1:** **General demographic information**.

	**Pre-puberty group**	**Middle-puberty group**	**Post-puberty group**
n	27	31	27
Age (y)	9.0 ± 0.6 (8.0−10.0)	13.5 ± 1.2 (12.0−15.0)	20.8 ± 1.9 (19.0−26.0)

*Mean ± standard deviation*.

### Stimuli

To prepare the visual stimuli, neutral and smiling facial expressions from the participants' mothers were recorded for approximately 3 min in a quiet experimental room prior to NIRS recordings, using digital video camera (GZ-MG40; Victor). To record a smiling face, the mother was asked to smile as if talking to her child: raise the corners of her mouth so that her smiling expression was most expressive, and look straight at the camera to provide eye contact stimuli (Ekman et al., 1990). To record a neutral face, the mother was asked not to show any facial expression. The video camera was positioned approximately 135 cm in front of the mother. Each video image was edited using Canopus Edius J (Thomson Canopus Co., Ltd., Japan) to obtain 30 s video stimuli of the mother with neutral and smiling expressions. To control physical characteristics among the mothers, each video image was edited using the following criteria: (1) the gaze was fixed approximately on the center; (2) the upper part of the body was visible; and (3) the image was recorded against a white background. Video stimuli were presented with no sound because of high variance in auditory information. To standardize facial expression across participants, several movie recordings were made, and the stimulus video created using recordings in which typical and defining smile features described by Ekman et al. (1990) were clearly visible.

### Procedure

#### Visual presentation task

During NIRS recordings, participants passively viewed videos of own (i.e., own condition) and unfamiliar (i.e., unfamiliar condition) mother's smiling. The video of smiling face was always preceded by the video of neutral expression of the same person. Their own mother's face was also used as an unfamiliar mother's face for another participant in the same developmental group. Thus, each mother's face was used once in the own and unfamiliar conditions. Video stimuli were presented on a monitor (17 in) located at a distance of approximately 50 cm from the participants.

After NIRS probe placement, each participant performed the visual presentation task with NIRS recordings taken. The task began with a blank screen showing a black background. A white hairline cross was presented for 30 s, and then the video stimulus of their own mother's neutral face was presented for 30 s as baseline period. Then, the video stimulus of the same mother's smiling face was presented for 30 s. After 30 s of a blank screen, a white hairline cross was again presented. Another video stimulus of an unfamiliar mother's neutral face was then presented for 30 s, followed by the same mother's smiling face. Finally, after 30 s, the unfamiliar mother's smiling face disappeared and 30 s of blank screen was presented.

For half of all participants, the unfamiliar mother's face was presented prior to the own mother's face to control residual presentation order effects. Participants had no task to perform and were asked to simply watch the video stimuli on the screen. None of the participants could identify the person presented as the unfamiliar mother.

#### NIRS recordings

During the visual presentation task, hemoglobin concentrations were measured at a sampling rate of 0.5 Hz using the 10-Ch NIRS system (NIRO-200; Hamamatsu Photonics, Japan; wavelengths 775, 810, and 850 nm, pathlength 18 cm). The modified Lambert–Beer law was used for calculating the oxyHb and deoxyhemoglobin (deoxyHb) concentration changes. NIRS probes were attached to the forehead according to the International 10–20 electrode system used in electroencephalography (EEG), such that a horizontal line through Fp1-Fpz-Fp2 matches the lowest two detectors in our NIRS system. Two emitters and eight detectors were aligned, as previously reported (Kida and Shinohara, 2013; Kida et al., 2014), resulting in 10 recording sites (channels). This position enabled assessment of the APFC. Our previous study using the same position (Kida and Shinohara, 2013) demonstrated spatial registration of the NIRS probe and channel locations using the NFRI toolbox (Okamoto and Dan, 2005; Singh et al., 2005) implemented in NIRS_SPM software (Ye et al., 2009), and identified the corresponding Brodmann's area.

#### Stimulus evaluation

After NIRS recordings, participants were asked to rate favorable impressions of own and unfamiliar faces on a visual analog scale (VAS) of 0–10 (0 = none and 10 = most), specifically, the happiness and comfort they felt while viewing the faces in each condition.

#### Salivary testosterone collection and analysis

Saliva samples were collected from each participant into small polypropylene tubes, during 11:00 and 14:00 on the day of the experiment to control for diurnal variation in testosterone concentrations. Saliva samples were frozen and stored at −80°C. Testosterone was assayed in duplicate by ELISA (Salimetrics, State College, USA). The intra-assay variation coefficient was 8.9%, while the inter-assay variation coefficients for high and low controls were 7.0 and 14.0%, respectively.

### NIRS data analysis

Before the statistical analysis, we identified the trials and channels that included artifactual fluctuation with sharp change of oxyHb and deoxyHb concentration. Following the precedence of the previous fNIRS studies (Peña et al., 2003; Takizawa et al., 2008), we identified waveform fluctuation as artifact whose point-to-point concentration change was larger than 5 μM/L·m. Note that this is more stringent criterion than those adopted in the previous studies (Peña et al., 2003; Takizawa et al., 2008). We further checked the results by visual inspection of the waveforms.

The data from participants whose data included artifacts in more than three channels were discarded from the final analysis. Other exclusion criteria included the failure to obtain mother's facial stimuli (3 participants), equipment failure (2 participants), and excessive head and bodily movement (12 participants). 17 participants were excluded from the analysis, resulting in 27 pre-puberty, 31 middle-puberty, and 27 post-puberty boys (Table 1).

Here, we mainly focus on the results based on oxyHb concentration changes, as we consider this the most sensitive parameter of hemodynamic responses (Malonek et al., 1997; Hoshi et al., 2001; Strangman et al., 2002; Shimada et al., 2005; Doi et al., 2013). At the same time, we also present deoxyHb results for completeness.

In quantifying the concentration change, mean concentration of oxyHb/deoxyHb during the last 20 s of the baseline was first calculated for each individual. Then, the concentration change was computed by subtracting the mean concentration during the baseline from that during the last 20 s of the smile presentation (Schroeter et al., 2004; Kida et al., 2014). All the statistical analyses were conducted with PASW Statistics 18.0 (SPSS Inc., IL USA).

## Results

### Concentration change of oxyHb

#### Overall developmental trend

Changes in oxyHb concentration in response to mother's smiling face were analyzed by Three-Way repeated measures analysis of variance (ANOVA) with channel (10 channels) and condition (own or unfamiliar) as within-participant factors, and developmental stage (pre-, middle-, or post-puberty group) as the between-participant factor. A significant interaction among the three factors [*F*_(18, 738)_ = 1.70, *p* = 0.03] was found, while the other main effects or interactions were not statistically significant (*p*s > 0.20). This indicates that there are differential patterns of neural activation to own and unfamiliar mother's smiles across the three age groups. To examine this Three-Way interaction, we performed a Two-Way repeated measures ANOVA separately for each developmental stage, with within-participant factors of channel (10 channels) and condition (own or unfamiliar).

#### Pre-puberty group

**Figure 1:**
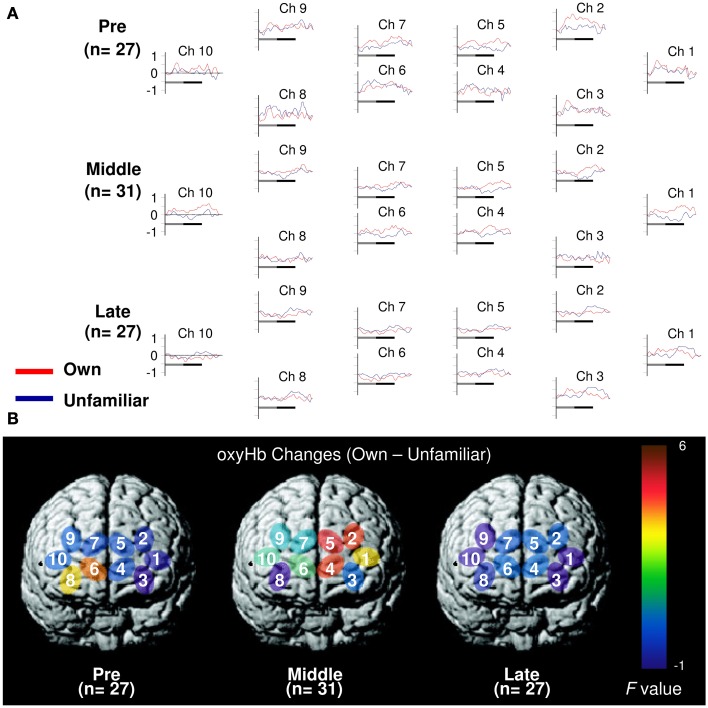
**Task-related oxyHb changes in the APFC. (A)** Time course during the neutral (shadow line) and smiling (solid line) face stimuli. Averaged waveforms activated by own (red) and unfamiliar (blue) conditions in the pre-puberty group (Pre), middle-puberty group (Middle), and post-puberty group (Post). **(B)** *F*-map of the response magnitude of oxyHb changes of own *vs*. unfamiliar conditions. Ch6 in Pre and Ch2, Ch4, and Ch5 in Middle reached significance.

**Figure 2:**
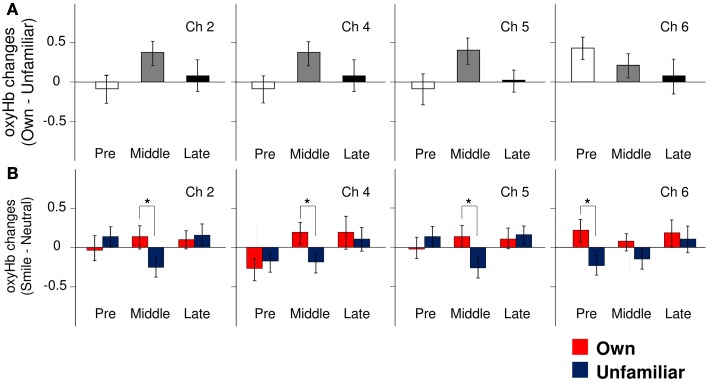
**The magnitude of oxyHb changes in the APFC. (A)** Group differences in the magnitude of oxyHb changes in response to viewing own-mother facial smiling against unfamiliar-mother facial smiling in Ch2, Ch4, Ch5, and Ch6. Error bars indicated SE. **(B)** Comparisons of the response magnitude of oxyHb changes (smiling minus neutral) in each condition (own: red bars, unfamiliar: blue bars) in Ch2, Ch4, Ch5, and Ch6. Error bars indicate SE. ^*^p < 0.05, vs. unfamiliar condition.

In the pre-puberty group (Figure 1A), the main effects of channel [*F*_(9, 234)_ = 1.50, *p* = 0.15] and condition [*F*_(1, 26)_ = 0.07, *p* = 0.79] were not significant, but there was a significant interaction between them [*F*_(9, 234)_ = 1.97, *p* = 0.04] (Figure 1B). Subsequent analysis showed that for Ch3 [*F*_(1, 260)_ = 5.87, *p* = 0.02], the unfamiliar condition exhibited a significantly greater oxyHb increase than the own condition, while for Ch6 [*F*_(1, 260)_ = 3.93, *p* = 0.05] (Figures 2A,B), the own condition yielded a significantly greater oxyHb increase than the unfamiliar condition. Furthermore, for Ch8 [*F*_(1, 260)_ = 2.90, *p* = 0.09], the oxyHb increase was marginally significant. No significant condition effect was found for the other channels (*p*s > 0.43) (Figure 1B). In summary, in the pre-puberty group, oxyHb increased in the right medial and inferior APFC in response to their own mother's smile compared with an unfamiliar mother. In contrast, in the left APFC, oxyHb increased more in response to an unfamiliar mother's smile than their own.

#### Middle-puberty group

In the middle-puberty group (Figure 1A), the main effect of channel [*F*_(9, 270)_ = 0.29, *p* = 0.98] was not significant, but condition was marginally significant [*F*_(1, 30)_ = 3.01, *p* = 0.09] and there was also a significant interaction [*F*_(9, 270)_ = 2.16, *p* = 0.03] (Figure 1B). Subsequent analysis showed that for Ch2 [*F*_(1, 300)_ = 4.95, *p* = 0.03], Ch4 [*F*_(1, 300)_ = 4.82, *p* = 0.03], and Ch5 [*F*_(1, 300)_ = 5.78, *p* = 0.02] (Figures 2A,B), the oxyHb increase for the own condition was significantly greater than for the unfamiliar condition. In addition, for Ch1 [*F*_(1, 300)_ = 2.89, *p* = 0.09], the oxyHb increase was marginally significant. No significant condition effect was found for the other channels (*ps > 0.16*) (Figure 1B). In summary, in the middle-puberty group, oxyHb increased more in the left APFC in response to their own mother's smile than an unfamiliar mother. Moreover, the activated region extended into the superior APFC.

#### Post-puberty group

In the post-puberty group (Figure 1A), there were no significant main effects for channel [*F*_(9, 234)_ = 0.12, *p* = 0.10] or condition [*F*_(1, 26)_ = 0.38, *p* = 0.54]. There was a marginally significant two-way interaction [*F*_(9, 234)_ = 1.71, *p* = 0.09] (Figures 1B, 2A,B).

### Concentration change of deoxyHb

**Figure 3:**
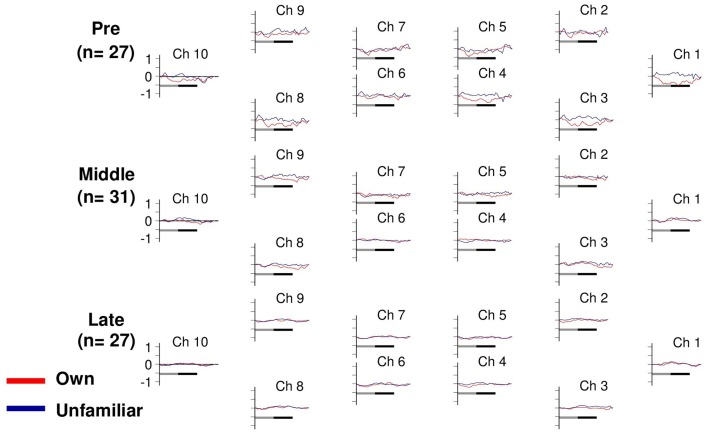
**Task-related deoxyHb changes in the APFC**. Time course during the neutral (shadow line) and smiling (solid line) face stimuli. Averaged waveforms activated by own (red) and unfamiliar (blue) conditions in the pre-puberty group (Pre), middle-puberty group (Middle), and post-puberty group (Post).

Changes in deoxyHb concentration were analyzed by Three-Way ANOVA with channel (10 channels) and condition (own or unfamiliar) as within-participant factors, and developmental stage (pre-, middle-, or post-puberty group) as the between-participant factor. Importantly, there was no significant interaction among the three factors [*F*_(18, 738)_ = 0.65, *p* = 0.86]. The other main effects or interactions did not reach significance, either (*p*s > 0.14) (Figure 3).

### Subjective ratings and salivary testosterone concentrations

Means and standard deviations for subjective ratings and testosterone concentrations are summarized in Table 2. Ratings for happiness and comfort are missing from three participants in the post-puberty group. The rating data from the remaining participants were analyzed using a Two-Way ANOVA with the between-participant factor of age and within-participant factor of condition (own–unfamiliar). Significantly higher ratings were found in the own than unfamiliar condition for favorable impression [*F*_(1, 82)_ = 204.37, *p* < 0.01], happiness [*F*_(1, 79)_ = 45.90, *p* < 0.01], and comfort ratings [*F*_(1, 79)_ = 67.25, *p* < 0.01].

**Table 2:** **Subjective ratings and Salivary testosterone concentration**.

		**Pre-puberty group**	**Middle-puberty group**	**Post-puberty group**
Favorable impression	Own: Unfamiliar:	7.7 ± 2.8 2.6 ± 2.0	6.5 ± 2.6 2.9 ± 2.1	7.7 ± 1.7 4.0 ± 1.7
Happiness	Own: Unfamiliar:	7.4 ± 2.2 5.4 ± 2.6	6.2 ± 2.3 4.2 ± 2.5	6.7 ± 1.7 4.8 ± 2.3
Comfort	Own: Unfamiliar:	7.7 ± 2.2 5.6 ± 2.4	6.5 ± 2.4 4.3 ± 2.5	7.3 ± 2.0 4.7 ± 2.3
Salivary testosterone concentration (pg/ml)		18.0 ± 10.2	67.0 ± 32.9	138.1 ± 49.0

*Mean ± standard deviation*.

We were unable to obtain a saliva sample from one participant in each age group. Salivary testosterone concentration from the remaining participants was analyzed by One-Way between-participant ANOVA with a factor of developmental stage. A significant main effect for age was found [*F*_(2, 79)_ = 79.69, *p* < 0.01]. Multiple comparisons revealed higher testosterone concentration in the post-puberty group than the middle- [*t*_(79)_ = 7.68, *p* < 0.01] and pre-puberty groups [*t*_(79)_ = 12.55, *p* < 0.01]. There was also a significant difference in testosterone concentration between the middle- and pre-puberty groups [*t*_(79)_ = 5.30, *p* < 0.01].

## Discussion

In the pre- and middle-puberty groups, the inferior and medial APFC was activated by their own mother's smile. However, different patterns of APFC activation were observed between the two groups. In the pre-puberty group, the right inferior and medial APFC (Ch6) was activated, whereas in the middle-puberty group, the activation pattern involved the left APFC (Ch4) and expanded into the superior APFC (Ch2 and Ch5). By contrast, there was no PFC activation in response to their own mother's smile in the post-puberty group. Importantly, concentration change of deoxyHb was not influenced by any of these factors, which rules out the possibility that the observed pattern of oxyHb concentration change derives from artifacts of bodily movement or facial muscle contraction. These findings indicate that as hypothesized, APFC activation to the primary attachment figure (i.e., own mother) changes dynamically around puberty.

### Pre-puberty

We found increased oxyHb in the inferior APFC in response to own mother's smile. The inferior and medial APFC is associated with reward processing (Kawabata and Zeki, 2004; Grabenhorst and Rolls, 2011). It has also been shown that a child's own mother smiling activates neural networks involved in reward processing (Kringelbach and Rolls, 2004; Kringelbach, 2005; Wallis, 2007). Thus, our present findings indicate that affective responses to own mother observed during infancy (Minagawa-Kawai et al., 2008) are sustained until at least 9 years of age.

In the pre-puberty group, we also observed increased oxyHb in response to unfamiliar mother's smiling in Ch3, which corresponds to the left superior frontal gyrus. Several existing studies have linked this regions to mnemonic encoding of verbal and visual materials (De Zubicaray et al., 2001; Tsukiura et al., 2002). Considering these, the increased activation to unfamiliar mother's face might reflect the willingness of pre-pubertal children to memorize the faces of friendly, smiling, person.

### Middle-puberty

In contrast to the pre-puberty period, we found increased activation in the left, but not right, APFC, which extended into the superior APFC. On the basis of the finding that reward expectation induces increased activation in the left prefrontal region (Ueda et al., 2003), our observed activation pattern may reflect positive maternal affect, as in pre-puberty children.

Previous studies have linked left superior APFC activation to empathy (Farrow et al., 2001) and autobiographical memory (Ryan et al., 2001). Therefore, it is possible that left superior APFC activation identified here reflects warm and empathic feelings toward their own mother, fostered by memory traces of nurturance and mutual experiences. This partially explains why a similar activation pattern was not observed in the pre-puberty group, who have relatively poor mnemonic capacity.

### Post-puberty

In post-puberty, the activation patterns observed during pre- and middle-puberty disappear, which presumably indicates that post-puberty boys do not show as high levels of maternal affection as younger boys. Possibly achievement of psychological independence from their parents and a stronger interest in romantic relationships (Csikszentmihalyi et al., 1977; Steinberger, 1989; Mendle et al., 2007; Koepke and Denissen, 2012) somehow weakens the strong affectionate response to mothers in this group.

### Limitations and future work

Although our study reveals pubertal developmental changes in APFC activation in response to a child's own mother, some methodological and theoretical limitations should be noted. First, NIRS with 10 channels was used, and we did not measure activation in cortical or subcortical regions other than the APFC. Previous neuroimaging studies have shown recruitment of various regions in addition to the APFC, in processing information on familiar people, including an individual's own mother (Ramasubbu et al., 2007). Therefore, it is without doubt that further study is required to clarify the full picture of the developmental course. In relation to this point, the relatively poor spatial resolution of NIRS prevented us from identifying the exact cortical location, which raises the possibility that our measured oxyHb increases may reflect a summation of different types of neural activation, e.g., excitatory and inhibitory activation, in diverse locations. Future, studies using other neuroimaging tools are required to determine the exact nature of the developmental pattern observed.

Second, we did not control the participants' facial expressions during NIRS recordings. It is possible that muscular movements associated with facial expressions may have produced artifacts in our NIRS data. Informal observation by video-recording of some of the participants have shown no signs of facial mimicry to smiling faces. Still, future studies using simultaneous measurement of facial electromyography to eliminate these potential artifacts are required (Schecklmann et al., 2010). Third, because of ethical concerns, we did not perform clinical examination of sexual maturity, a gold standard for pubertal development (Marshall and Tanner, 1970). Because of this, we cannot exclude the possibility that each age group is heterogeneous from the perspective of physiological development. The finding that the middle-puberty group showed intermediate levels of testosterone concentration, partially validates our grouping at least in the group level. Nevertheless, the present results should be replicated with application of a more rigorous sexual maturity criteria in order to fully exclude the possibility that pre-pubertal child was included in the middle puberty group.

Fourth, we did not perform standardized assessment of the mother–child relationship, which makes it difficult to link our findings to the existing literature on qualitative changes in the pubertal attachment relationship. In relation to this point, the mother's smile may be interpreted as a harbinger for mental agony for children who suffer from an abusive relationship with their mother. Although our subjective evaluation indicated that the present participants generally had a positive attitude and felt intimacy toward their mothers, the relationship between the qualitative aspect of the mother–child relationship and neural activation pattern is an interesting topic for future research. Finally, our study involved a cross-sectional design with three different developmental stages. Thus, morphological or endocrine differences for each individual were unavoidable. Future, longitudinal studies are required to provide further support that the neural basis for attachment-related positive affect in pubertal boys correlates with pubertal brain development stage.

## Conclusion

In conclusion, our study provides the first evidence that in boys, APFC activation in response to their mother's smile changes during pubertal development. These changes may result from brain activation reflecting detection of a (salient) stimulus, such as their own mother's smile. The methods used in this study may be further validated for efficacy as a biomarker to detect vulnerability of various functions (such as reorganization of one's identity and self-perception or inter-personal relations) that develop based on mother–child communication by studying abused or maltreated children.

### Conflict of interest statement

The authors declare that the research was conducted in the absence of any commercial or financial relationships that could be construed as a potential conflict of interest.
